# Bonney's Rapid Triple Stain

**Published:** 1908-10-17

**Authors:** 


### Clinical Methods.

### BONNEY'S RAPID TRIPLE STAIN.

Bonney's triple stain is intended, not for blood films, but for histological preparations of normal and pathological tissues. It has been considerably simplified since it was originally published in 1906; and its newest form is described in the thirteenth volume of the *Archives of the Middlesex Hospital.*

In the original stain, Safranin, Methyl-Violet, and Orange-G were amalgamated in such a way that the Safranin selected the cytoplasm of the cells, the Methyl-Violet their chromatin (nuclei), and the Orange-G the interstitial fibrous tissue. Pyronin is now substituted for Safranin, and the following stock solutions are required:

1. *Methyl-Violet—Pyronin Solution.*—Methyl-Violet, 0.25 gramme; Pyronin, 1 gramme; distilled water to 100 c.c. The dyes are dissolved in the water by heating, and the product is filtered off, labelled, and stored.

2. *Acetone-Orange-G Solution.*—A 2 per cent. aqueous solution of Orange-G is made by heat, and filtered. The solution is then added drop by drop, and quite slowly, to 100 c.c. of acetone. The mixture should be kept well stirred with a glass rod the while. When the fluid has attained a pale yellow colour a faint cloudiness appears. Further addition of the stain increases this until a flocculent precipitate is formed. If the addition of Orange-G is still continued, drop by drop, the precipitate presently redissolves; immediately it has done so the solution should be filtered off into the stock bottle. Dr. Bonney lays particular stress upon the necessity of adding the Orange-G solution very slowly, for otherwise the precipitate does not appear. The importance of the precipitate appears to be that it is a guide in knowing when enough Orange-G has been added. After twenty-four hours a crystalline precipitate appears in the bottle, and this increases with time, apparently without impairing the efficacy of the solution. After a few weeks, however, it may be advisable to add a few drops more of the watery solution of Orange-G.

### Technique.

The material to be stained cannot be fixed either by means of chromates or by formalin; these render the method useless unless subsequent to staining the sections are treated by oxidising agents. The tissue should be fixed either in alcohol, or in sublimate solution, or in acetic alcohol made up of two parts of absolute alcohol and one part of glacial acetic acid of freezing point between 14.7° C. and 15° C.

The section is stained for two minutes in the Methyl-Violet—Pyronin solution, then rapidly washed in water, after which all superfluous water is wiped from the slide as far as possible and excess of Acetone-Orange-G is poured on. A cloud of colour comes out. The stain is allowed to drain quickly off, and a fresh quantity of it is poured over the slide, and this process is repeated until no more colour comes out of the section. The latter is then rapidly washed in pure acetone, all precautions being taken against the volatile acetone evaporating so fast as to leave the section dry. The acetone is replaced by xylol to clear the tissue, and the preparation is mounted in Canada balsam in the usual way. The whole process of staining should not occupy more than five minutes. It is possible to correct for over or understaining by returning the tissue through acetone to water, and commencing the staining again. The chromatic substance of nuclei, mitotic figures and nucleoli take up the Methyl-Violet; so also does keratin. The cytoplasm of cells stains varying shades of red with the Pyronin. The interstitial connective tissue and the intracellular matrices stain yellow with the Orange-G.

It is stated that the tissues retain their colour permanently; time, however, is needed before this can be decided with certainty. Meanwhile the colour differentiation of the individual elements of the tissues is stated to be so excellent that this simplified Bonney's triple stain would seem to merit an extensive trial in histological work.

